# The impact of urbanization on health depends on the health metric, life stage and level of urbanization: a global meta-analysis on avian species

**DOI:** 10.1098/rspb.2024.0617

**Published:** 2024-07-17

**Authors:** Rachel Reid, Pablo Capilla-Lasheras, Yacob Haddou, Jelle Boonekamp, Davide M. Dominoni

**Affiliations:** ^1^ School of Biodiversity, One Health and Veterinary Medicine, University of Glasgow, Graham Kerr Building, 82 Hillhead Street, Glasgow G12 8QQ, UK

**Keywords:** urban ecology, avian populations, health biomarkers

## Abstract

Stressors associated with urban habitats have been linked to poor wildlife health but whether a general negative relationship between urbanization and animal health can be affirmed is unclear. We conducted a meta-analysis of avian literature to test whether health biomarkers differed on average between urban and non-urban environments, and whether there are systematic differences across species, biomarkers, life stages and species traits. Our dataset included 644 effect sizes derived from 112 articles published between 1989 and 2022, on 51 bird species. First, we showed that there was no clear impact of urbanization on health when we categorized the sampling locations as urban or non-urban. However, we did find a small negative effect of urbanization on health when this dichotomous variable was replaced by a quantitative variable representing the degree of urbanization at each location. Second, we showed that the effect of urbanization on avian health was dependent on the type of health biomarker measured as well as the individual life stage, with young individuals being more negatively affected. Our comprehensive analysis calls for future studies to disentangle specific urban-related drivers of health that might be obscured in categorical urban versus non-urban comparisons.

## 1. Introduction

Urbanization is characterized by profound modifications of natural habitats, which includes the creation of impervious surfaces and buildings [1,2], the introduction of high levels of chemicals and metals in the ground [3,4], increased levels of air [2,5], noise and light pollution [6,7], high amounts of refuse, including anthropogenic food sources [8,9], and increased ambient temperatures (the ‘heat island effect’) [1]. In humans, many of these environmental factors have been associated with changes in physiological processes linked to health, which have been discussed in great detail in a variety of reviews, including those by Mabahwi *et al*. and Tong *et al*. [10,11].

Several studies have suggested that these environmental factors associated to urbanization may not only affect human but also affect wildlife health [12]. Noise pollution has been shown to impair reproduction and territorial communication of wild species [13], and one of the suggested mechanisms for this is an increase in the concentration of stress hormones and inflammatory molecules [14]. Similarly, artificial light at night has been shown to disrupt circadian rhythms, including that of several physiological processes, as well as increase activity and metabolic rate, which may have consequences for health [15,16]. An increase in chemical and metal pollution has also been associated to downstream health and reproductive consequences [17]. The anthropogenic food that is readily available in urban environments, either directly or indirectly provided to wildlife by humans, often lacks many essential macro- and micronutrients, which may result in poor diet and lead to knock-on effects on oxidative stress, gut microbiome [18], immunity [19] and infection risk [20].

Because of the profound effects that each urban environmental factor may have on organismal processes linked to health, there are many studies that show that wildlife populations living in urban habitats are in worse health than their non-urban conspecifics. This is particularly true for birds, one of the most studied animal taxa in urban ecology. Urban great tit (*Parus major*) nestlings have been shown to be smaller and in poorer condition than non-urban chicks [21–23]. Increasing levels of urbanization have been shown to lead to higher feather corticosterone levels in juvenile house sparrows (*Passer domesticus*), which may constrain their development [24]. Transcriptomics studies have shown that urban blue tits (*Cyanistes careuleus*) and great tits have higher levels of gene transcripts associated to inflammatory responses, compared with their non-urban cousins [19,25]. However, not all studies support the idea that urbanization invariably and negatively affects health. In song sparrow (*Melospiza melodia*), increased levels of urbanization had no impact on their stress physiology or body condition [26]. Another study found that non-urban burrowing owls (*Athene cunicularia*) actually had higher levels of stress- induced corticosterone when compared with urban birds, and suggested this could be owing to urban birds having adapted to deal with stress more effectively [27]. In two Australian passerines, coccidian infection increases with increasing urbanization in red-brown finches (*Neochima temporalis*), but not in superb fairy wrens (*Malurus cyaneus*) [28]. In black sparrowhawks (*Accipiter melanoleucus*), urbanization was negatively associated with some biomarkers of immunity and oxidative stress, while the majority of such markers were not affected [29]. Similarly, a large comparative analysis on the prevalence of *Salmonella* and *Campylobacter* in birds, two avian gastrointestinal bacteria, showed that while *Salmonella* prevalence was mostly affected by ecological factors including urbanization where prevalence was higher in urban areas, *Campylobacter* prevalence was largely associated with life-history traits [30].

There are many factors that may impact the relationship between urbanization and health, for example the early developmental stage for many bird species is a particularly sensitive time, and indeed a large proportion of the mortality in birds occur in early life [31–33]. Therefore, negative impacts of urbanization on health may be more apparent in younger birds than adults. A few studies have shown this with nestling/juvenile birds being in poorer fitness and condition in urban areas while adults of the same species appear to be less affected [34–36]. However, studies have also shown no age-specific urban effects on avian health markers [23,37]. A comprehensive analysis of the literature is thus needed to assess the evidence for general age-dependent urban effects on health. Urbanization may also impact different bird species in different ways, for instance because the way a species responds to urban stressors may depend on specific traits, which may either help them to adapt or limit their ability to adapt to such stressors [38], yet no study to date has looked into this at a global scale. Geographic location and specifically latitude can also be a factor on how urbanization impacts health. Recent rates of urbanization vastly differ between different regions of the world [39], with faster rates of urbanization in tropical areas [40,41]. This means that animals living in different cities around the world have been exposed to different rates of urban-related environmental change in the last decades, and this may affect their ability to cope with such changes and as a result impact their health [42].

Taken all together, while previous research has suggested that urbanization may impact upon the health of wild avian species, the evidence remains inconclusive, and rather suggests that urban-related health change may be dependent on the studied species, the age of the individuals sampled and location of the study. Moreover, results also strongly differ depending on which health biomarker was measured, and most studies focused only on one or few of such markers. Thus, there is a need to assess the effect of urbanization on wildlife health by synthesizing and analysing the results of existing literature at the global scale, across all avian species, age classes and biomarkers of health. Here, we build on the work of previous meta-analyses that have investigated the relationship between urbanization and health. These previous works found contrasting results but overall suggests there is a relationship between urbanization and health and that this relationship may depend on a variety of factors including the study species and health biomarker measured [43–47]. Crucially, an important part of our approach was that we quantified the level of urbanization at each location included in our meta-analysis, while sites were traditionally categorized as urban versus non-urban. Urban areas can be spatially heterogeneous and thus the level of urbanization at each sampled location may strongly influence the difference between urban and non-urban populations [48]. Using the level of urbanization as a continuous variable in a meta-regression analysis may help us to uncover any hidden effects that we may not see when we group all urban areas in one category [48]. Moreover, we also included a wider range of health biomarkers than what has been used previously. We believe that this study will be of importance to policymakers as identifying the contexts in which urbanization impacts wildlife health is crucial to understand what areas need to be directly targeted for conservation management. This includes a better understanding of what species are more sensitive to urbanization and why, whether certain life stages are more at risk, as well as what health metrics are most likely to be impacted by urbanization (and therefore could be prioritized as relevant biomarkers). Such understanding would be crucial to protect and enhance urban biodiversity in a world that is projected to become increasingly more urbanized.

We performed multiple meta-analyses to increase our understanding of the complex relationship between urbanization and health in birds. We aimed to inform urban ecology theory as well as to guide future urban planning and management efforts. The questions we specifically want to answer are:

— Is there an overall impact of urbanization on bird health? We would expect that there will be an overall negative effect of urbanization on bird health as it is well established that urban areas have many novel stressors that may impact health [12,14,49,50].
— Is there a relationship between the degree of urbanization and health? We expect to find a stronger negative relationship between urbanization and health when the level of urbanization increases, an increase in urbanization will likely mean an increase in urban stressors that have the potential to impact health.
— Are specific health traits more sensitive to urbanization? We predict that certain health traits are likely to be more sensitive to urbanization as several studies have found strong effects of urbanization on health traits such as telomere shortening [23,37,51], other studies have also found contrasting results on the same health trait, for example evidence for effects on corticosterone have been shown to be more variable [24,26]. This could suggest the relationship will be dependent on other variables.
— Is the relationship between urbanization and health dependent on species-specific traits including maximum lifespan, trophic niche, migratory behaviour, primary behaviour and life stage? We expect that there will be an effect of species-specific traits on this relationship. Studies have shown that having specific traits can either help a species adapt or will hinder their ability to adapt to urban environments [36]. For example, it has been shown that nestlings will be more sensitive to environmental change than adults, so we may find a stronger effect of urbanization on health in this particular life stage [31,32,34]. Species that have certain trophic niches may also be more sensitive to urbanization, for example we may find that insectivores are in poorer health owing to reduced food availability [52].
— Is the relationship between urbanization and health dependent on latitude? We expect that there will be an effect of latitude on the relationship between urbanization and health. Rates of urbanization vastly differ around the world, and species that inhabit regions where the rates of urbanization are increasing rapidly may be more sensitive to these changes [42,53].

## 2. Methods

### (a) Literature search

We first created a search string using keywords that are relevant to the study, selected by reading relevant literature, the search string which we used for the meta-analysis is shown below:

(‘City*’ OR ‘Urban*”) AND (‘Aves’ OR ‘Avian’ OR ‘Bird*’ OR ‘Ornithol*’ OR ‘Passerine*’ OR ‘Passeriform*’ OR ‘Songbird*”) AND (‘Body condition’ OR ‘Corticosterone*’ OR ‘CORT*’ OR ‘Disease*’ OR ‘Infection*’ OR ‘Immun*’ OR ‘Oxidative stress’ OR ‘Parasite*’ OR ‘Stress*’ OR ‘Telom*’ OR ‘Telomere attrition’ OR ‘Telomere shortening’)

This search string was used to search for relevant peer-reviewed published papers on the Web of Science core collection. All the literature that was flagged using this search string was then extracted and we de-duplicated the initial search results in Excel using the Excel duplicate tool. We recorded number of results for each search iteration following Preferred Reporting Items for Systematic Reviews and Meta-Analysis (PRISMA) guidelines [54]. The PRISMA diagram for the meta-analysis is shown in electronic supplementary material, figure S1.

### (b) Inclusion criteria

**Figure 1.**
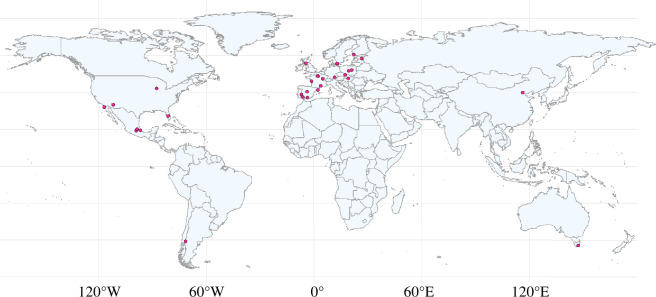
World map representing the geographic coverage of our study. The pink circles depict the locations where coordinates were known and could be extracted to calculate urban score.

To be included in the meta-analysis, studies must have been comparing paired urban and non-urban populations. In the case of a study where the sampling design was an urbanization gradient, then we used data extracted from the populations located at the urban and non-urban extremes of the gradient which was the case for 45 studies. Four studies used multiple urban and non-urban sites not really representing a gradient, and in these cases, any descriptions of the sites presented were used to extract data from the most urban and non-urban populations. If this was not possible, a random number generator was used to randomly select a non-urban and urban population to be included in the analysis. Studies were only included if the focal species they were measuring belonged to the avian class. The study must also have been measuring at least one health biomarker which we defined as any physiological or morphological measures or a measure of parasitism or disease that could have an overall effect on an organism’s fitness and survival. We first screened all studies by reading the title and the abstract, marking all relevant studies based on whether or not they met these inclusion criteria (electronic supplementary material, figure S1). After inspecting 4133 studies published between 1981 and 2022 (electronic supplementary material, figure S1), our meta-analysis included 644 urban-non-urban paired comparisons from 112 studies published between 1989 and 2022 from 16 health biomarkers (described in electronic supplementary material, tables S2 and S3) and included 51 bird species shown in electronic supplementary material, figure S1 [21,23,24,26,34,35,37,55–152] (figure 1).

### (c) Data extraction

We read all relevant studies (*n* = 217; electronic supplementary material, figure S1) in full. We then extracted any qualitative information about the study, including the focal species, health trait, authors, title, publication year, publishing journal, country the study took place in and how many years the study took place over. We also extracted information about the study location, and specifically about the geographic coordinates, if recorded. We then extracted the quantitative data needed to calculate effect sizes for each paired urban and non-urban locations, which included the standard deviation, the mean and the sample size of the health biomarker measured for each population. This was done in different ways depending on the study. For most studies, we extracted data from tables or text. In some cases, we extracted data from figures using the function ‘metaDigitise’ in the ‘metaDigitise’ (v. 1.0.1) [153] package in RStudio [154]. We also extracted data from the electronic supplementary materials or directly from the raw data that were provided as part of the publication. When the study had missing data or did not report the information needed to calculate effect sizes, we contacted the corresponding author recorded on the study to request the missing data. The authors who responded and sent data that were used in the meta-analysis are shown in electronic supplementary material, table S1.

To calculate the standardized mean difference between paired urban and non-urban locations for the health traits measured in each study, we calculated Hedges's *g* [155] as well as the sampling variance of the effect sizes, using the function ‘escalc’ in the ‘Metafor’ package (v. 4.4.0) [156]. To allow interpretation of the outcome of the meta-analysis, we assigned a direction of effect for each of the health traits included in the study. Specifically, if an increase in the value of a given health trait would have a predicted negative impact on health in urban environments, then this would be considered as a negative direction. The effect sizes for all health traits with a negative direction were flipped by multiplying them by −1. If a health trait was too complex to determine directionality, then this was excluded, this included biomarkers such as measures of gut microbiome, certain hormones (testosterone and oestradiol) as well as gene expression data. The directionality of each health trait is shown in electronic supplementary material, table S2.

### (d) Phylogeny

We extracted phylogenetic trees from the Open Tree of Life (https://opentreeoflife.github.io), using the interface provided by the R package ‘rotl’ (v. 3.1.0) [157]. We calculated tree branch length, built a phylogenetic correlation matrix and included this in all phylogenetic multilevel meta-analytic models. The phylogenetic signal was assessed in the meta-analysis based on the proportion of variation explained by phylogeny. The phylogenetic tree used for analysis is shown in electronic supplementary material, figure S2.

### (e) Additional variables

We also included additional variables to investigate the mechanisms mediating urban effects on avian health. We included species-specific traits such as trophic niche (the major resource types used), primary behaviour (the dominant locomotory behaviour while foraging) and migratory behaviour, which we extracted from the AVONET database [158] (electronic supplementary material, table S3). The maximum lifespan of the species was also included as a moderator and was extracted from the AnAge database [159]. Other moderators included the life stage (nestling or juvenile/adult) and the latitude of the study area (electronic supplementary material, table S3).

### (f) Urban score

For the subset of the studies where exact coordinates were reported for both the non-urban and urban sites, or where the corresponding authors had provided this information upon request, we calculated the degree of urbanization of each site, hereafter named ‘urban score’ using previously developed methods by Capilla-Lasheras *et al*. [48]. We did so by using the Copernicus Climate Change Service ICDR Land Cover data [160], which provides consistent land cover per year with a global coverage and spatial resolution of circa 300 m per pixel. Each pixel is classified as 1 of 22 land cover categories which are defined by United Nations Food and Agricultural Organization Land Cover Classification System.

We first extracted the number of pixels belonging to each land cover category within a circular buffer around each urban and non-urban location. We performed this operation for each of 13 buffer radii from 150 to 5000 m, in intervals of 250 m. We calculated the urban score as the proportion of each buffer area that was categorized as urban land cover type [160]. We then verified that urban score was higher in the urban than in the non-urban location which was the case for each paired location.

### (g) Data analysis

First, to evaluate the general effect of urbanization on bird health, we fitted a phylogenetic multi-level (intercept only) meta-analysis with Hedges g as the response term. Second, to test whether the effect of urbanization on health depended on the specific health trait considered, we ran another multi-level meta-analysis that included health trait as a moderator. Third, to investigate whether differences in health may be better captured by the level of urbanization rather than a simple dichotomous urban/non-urban variable, we ran phylogenetic meta-regression models to explain differences in health between urban and non-urban populations (i.e. Hedges's *g*), where the difference in urban score between the two populations were included as a continuous moderator (hereafter, ‘urban score’). We ran these models for every buffer radius from 250 to 5000 m at 250 m intervals, and then performed model selection to identify the model with the best fit which was determined to be the model with the lowest Akaike information criterion (AIC) value. This was found to be the model at 1000 m buffer radius. The results of this model were used for biological inference. The AIC values for models ran at each spatial scale can be found in electronic supplementary material, table S4. The 1000 m radius should be representative of the breeding home range of most species included in our study. We also ran this same model without urban score as a moderator to test whether the inclusion of urban score increased model fit. However, urban score was not used in the subsequent models as this information was only available for a small subset of the effect sizes used in the meta-analysis.

Fourth, to investigate whether species traits or latitude would modulate the effect of urbanization on health, we ran another multi-level meta-analysis which included life stage, primary behaviour, trophic niche, migratory behaviour and latitude (as an absolute value) as moderators. A separate model was then run with maximum lifespan as a moderator as this information was not available for all of the effect sizes.

Last, since several biomarkers were measured either on nestlings or adult birds, we decided to subset the data by life stage and then, we ran independent analyses on each subset using health trait as a categorical moderator.

We included study ID and phylogeny as random effects in each model as well as an unique ID for each individual effect size to account for residual variation owing to multiple observations from the same studies, but only in the models where health trait was not included as a fixed effect (as only one observation per health trait and study was included in our models). We calculated the heterogeneity for each of the phylogenetic multi-level models using the ‘i2_ml’ function from the OrchaRd package (v. 2.0) [161]. This allows us to calculate the percentage of total relative heterogeneity as well as the heterogeneity explained by each random effect included in the model.

We then checked the data for any signs of publication bias. This included looking for any signs of small study effects as well as time lag bias. Small study effects occur when smaller studies show different, in many cases, larger treatment effects than larger studies. Time lag bias may occur when more statistically significant effects are published quicker than smaller or non-statistically significant effects [12–14,49]. To test for bias, we ran two extra multi-level meta-regressions with the same random structure as the previous models but using a moderator that was either: (i) the square root of inverse sampling variance to test for small study bias [162] or (ii) year of study publication mean-centred for time lag bias [162,163]. The *R*^2^ was then calculated using the ‘R^2^_ml’ function from the ‘OrchaRd’ package (v. 2.0) [161]. This was done for the main dataset as well as for any subset data that were used for subsequent analysis.

All data processing and analysis were completed using R (v. 4.2.1) [154].

## 3. Results

### (a) Does urbanization impact avian health?

**Figure 2.**
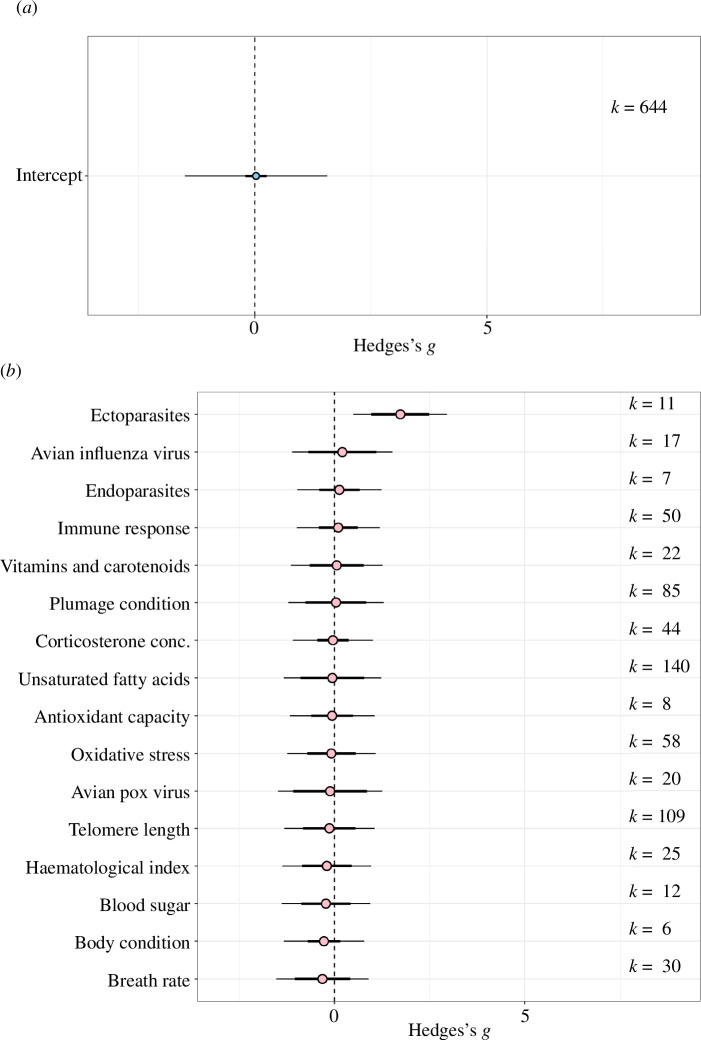
Urbanization has no overall impact on avian health and most avian health traits are not affected by urbanization. (*a*) Results from the intercept only meta-analytic model. The estimated health impact is reflected in the effect size (Hedges's *g*) which is displayed on the *x*-axis. Model estimates for Hedges's *g* is shown along with its 95% CIs (thick whisker), 95% prediction intervals are also shown (thin whisker). (*b*) Second meta-analytic model where health trait was a moderator. The orchard plot shows different biomarkers of health and their estimated impact on the overall direction of health in urban environments. The health impact is reflected in the effect size (Hedges's *g*) which is displayed on the *x*-axis. The health biomarkers that were measured are displayed on the *y*-axis. Positive estimates assume a positive health consequence in urban bird populations. *k* represents the corresponding number of effect sizes. Full model outputs are shown in electronic supplementary material, table S5.

Overall, we did not detect any significant impact of urbanization on avian health (mean estimate [95% confidence interval, CI] = 0.0276 [−0.1992, 0.544]; figure 2*a*). Total heterogeneity was high (*I*^2^ = 89.63%) with 4.34% of it explained by phylogeny, while 18.28% of it was explained by differences between studies. The second model, which was run with health trait as a moderator showed that health, as determined by lower load of ectoparasites, was higher in urban environments (mean estimate [95% CI] = 1.728 [0.963, 2.493]; figure 2*b*). This suggests birds had fewer ectoparasites in urban environments. Full model outputs are shown in electronic supplementary material, table S5.

**Figure 3.**
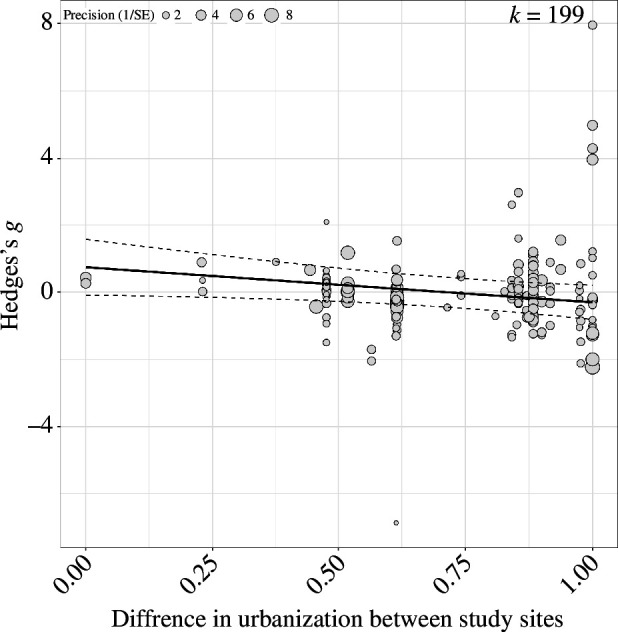
Larger differences in urbanization lead to stronger effect on avian health. The plot shows the effect of the difference in urban score between paired non-urban and urban populations on the health of bird species. The differences in urban score between two paired study sites is shown on the *x*-axis, while Hedges's *g* is shown on the *y*-axis. The individual effect sizes are scaled by their precision which is (1/SE), the more precise the effect size is the larger the circle. The solid line represents the model estimate, with the dashed lines representing the 95% CIs. *k* shows the total number of effect sizes.

We found a significant negative relationship between urban score and overall health (mean estimate [95% CI] = −1.042 [−1.992, −0.092]; figure 3). The regression line crosses 0 at an urban difference of 0.75, however, the slope was negative indicating that the urban effect is more negative the more urbanized a population is. The AIC score of the model without urban score as a moderator was 2775 and the AIC score of the model when urban score was included as a moderator was 2707, therefore the model was a much better fit when urban score was included as a moderator. The effect of urban score and overall health at each spatial scale is shown in electronic supplementary material, figure S3.

### (b) Is the relationship between urbanization and bird health impacted by species traits, latitude or life stage?

**Figure 4.**
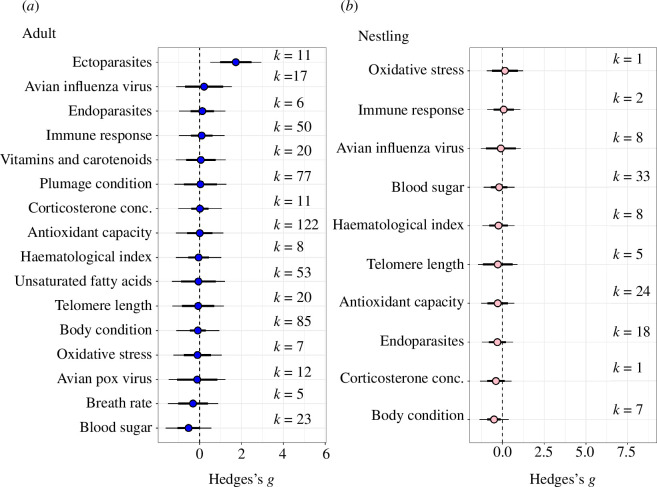
The relationship between urbanization and specific health traits depends on the life stage. This orchard plot shows different biomarkers of health and their assumed impact on the overall direction of health in urban environments for (*a*) adults and (*b*) nestlings. *k* represents the corresponding number of effect sizes. Full model outputs are shown in electronic supplementary material, tables S6 and S7.

The maximum lifespan of bird species did not have a significant impact on the relationship between urbanization and health (mean estimate [95% CI] = 0.011 [−0.565, 0.543]; electronic supplementary material, figure S5). Similarly, trophic niche, migratory behaviour and primary behaviour did not affect the relationship between urbanization and health (electronic supplementary material, figure S4). Latitude was also found not to affect relationship between urbanization and health (mean estimate [95% CI] = −0.005 [−0.02, 0.001]; electronic supplementary material, figure S6). The majority of studies included in this meta-analysis were located in the Northern Hemisphere (electronic supplementary material, figure S6). Finally, life stage of birds does not appear to have a significant effect on their overall health in urban environments (adult data subset: mean estimate [95% CI] = −0.191 [−1.079, 1.154], nestling data subset: mean estimate [95% CI] = −0.132 [−1.418, 1.154]; figure 4*a*).

### (c) Decomposing the relationship between life stage, urbanization and health

While life stage did not seem to have a significant effect on the relationship between urbanization and health, it did appear that nestling health was negatively associated to urbanization, while adult health was not (electronic supplementary material, figure S4*a*). This, together with the fact that many health traits were not analysed in both adults and nestlings, prompted us to run two further models to decompose effects separately for adults and nestlings. We found that adult birds have less ectoparasite burden in urban environments compared to non-urban environments (mean estimate [95% CI] = 1.728 [0.972, 2.484]; figure 4*a*), while ectoparasite burden was never measured in nestling birds. Adult birds also tended to have higher blood sugar levels in urban environments, but this was not a significant relationship (mean estimate [95% CI] = −0.53 [−1.064, 0.005]; figure 4*a*). Unlike adults, whose body condition did not differ between habitats, nestlings were found to be in worse body condition in urban than non-urban environments (mean estimate [95% CI] = −0.499 [−0.901, −0.097]; figure 4*b*). To explore this effect further, we ran an additional meta-analysis with effect sizes of nestling body condition as response variable, and trophic niche and primary behaviour as moderators. There does not appear to be a significant effect of species trophic niche on nestling body condition in urban areas (electronic supplementary material, figure S7*a*). Birds with a terrestrial primary behaviour are in worse body condition in urban environments when compared to non-urban areas (model estimate [95% CI] = −0.9461 [−1.6854,−0.2067]; electronic supplementary material S5*b*). Birds with an insessorial primary behaviour also appear to be in worse body condition in urban areas but this relationship was not significant (mean estimate [95% CI] = −0.4487 [−0.9142, 0.0169]). Generalist species did not appear to have their health impacted by urbanization (mean estimate [95% CI] = −0.515[−0.8043, 0.5012]). Full model outputs are shown in electronic supplementary material, tables S7, S6 and S8.

### (d) Publication bias

We found no evidence of small study effects in the main dataset (mean estimate [95% CI] = −0.0482 [−0.565, 0.543]; electronic supplementary material, figure S8) or time lag bias (mean estimate [95% CI] = 0.007 [−0.01, 0.024]). We also found no evidence of small study effects or timelag bias (mean estimate [95% CI] = 0.0002 [−0.018, 0.019]; electronic supplementary material, figure S9*a*) in the data subset by ‘adult’. There was also no evidence of small study bias in the nestling subset dataset (mean estimate [95% CI] = −0.446 [−2,294, 1.402]; electronic supplementary material, figure S9*b*). However, we did find evidence of time lag bias in the nestling subset dataset (mean estimate [95% CI] = 0.064 [0.003, 0.126]). There was no evidence of small study bias (mean estimate [95% CI] = −0.362 [−1.906, 1.183]) or time lag bias (mean estimate [95% CI] = 0.031 [−0.021, 0.083]) in the subset of data where urban score was calculated.

## 4. Discussion

We compiled a global dataset of avian health biomarkers including physiological and morphological biomarkers as well as measures of disease and parasitism for paired urban and non-urban populations, to provide a holistic view on how urbanization impacts avian health. A phylogenetically controlled meta-analysis revealed that urbanization did not show a clear relationship with overall health. However, when the degree of urbanization, rather than a dichotomous urban–non-urban category was used in the models, avian health did appear to decline with increasing urbanization, suggesting this quantitative method is more sensitive. We then explored these patterns in detail and found that the effect of urbanization on avian health was dependent on both the health biomarker measured as well as the life stage of the bird population. The meta-analysis revealed that adult birds inhabiting urban environments had reduced ectoparasite burden and slightly higher blood sugar levels while nestlings living in urban environments tended to be in worse body condition.

It is well documented in the literature that organisms inhabiting urban environments can face negative implications to their health and wellbeing [19,29,73]. Our results indicate that there is no significant effect of urbanization on overall avian health. Moreover, our analysis showed a high total heterogeneity in Hedges's *g*. These findings indicate large variation among both studies and species in how urbanization associates with changes in health biomarkers. While the lack of an overall difference in health between urban and non-urban birds might seem at first puzzling, our follow-up analysis that considered the urban score of each location, and specifically the difference in urban score between paired urban and non-urban locations, revealed a different picture. The effect on health seemed to reach 0 when the difference in urban score was high, which may suggest that we might see negative impacts only at very high levels of urbanization. In addition or alternatively, the low levels of ectoparasite burden in urban areas could have balanced out the otherwise negative impact of urbanization on health.

Regardless of the mechanistic explanation, this result does suggest that urban score may be a better predictor of avian health than a simple dichotomous classification of urban and non-urban populations. Cities are a mosaic of heterogeneous habitats that vary greatly from each other in their abiotic and biotic characteristics, including levels of pollution (light, noise, chemical and metal) [2,5–7], human population density [164], amount and quality of green space [1,2,164], degree of anthropogenic food provisioning [8,9], presence of invasive species [165]. Recent work suggests that although the degree of urbanization can account for a large proportion of the variance found between habitats [166–168], it still may not be enough to describe more complex relationships. Instead, quantifying the impact of urban-specific environmental factors may be more revealing. This approach could be beneficial for future studies (see for instance [169]), including in meta-analyses [170].

Our follow up analyses revealed some mechanisms by which avian health may be affected by urbanization. We found that birds had a reduced ectoparasite burden when they lived in urban areas in comparison to their non-urban conspecifics, which could have positive health outcomes. This result could be owing to urban areas having a warmer micro-climate which has been shown to reduce tick numbers [171]. However, a warmer micro-climate has also been shown to increase mosquito numbers, as these can feed and reproduce faster when it is warmer. Thus, this effect is probably dependent on the ectoparasite species being measured [172]. Adult birds in urban areas may also spend less time foraging owing to availability of supplemental food sources. This could potentially mean they have more time for behaviours that would allow them to reduce their ectoparasite burden such as preening [173]. Previous work suggested that *Ixodes* ticks are studied more than any other arthropod parasite, likely owing to them being easy to detect on a host through visual examination [172], and that most studies on avian ectoparasites are clustered in Europe and North America on urban-adapted or introduced bird species [172]. The relationship shown in our current study is drawn mostly from studies looking into the burden of ticks on adult blackbirds in urban and non-urban populations in Europe [104,124,142]. This limits our ability to make general assumptions about the relationship between urbanization and ectoparasite burden on avian species and calls for future studies to look into other bird–parasite relationships in urban areas.

Our analysis showed that migratory behaviour, primary behaviour, trophic niche or maximum lifespan did not affect the relationship between urbanization and health in bird species. The ability of species to cope with and adapt to environmental changes such as urbanization can be dependent on species traits such as their diet, dispersal ability and behavioural flexibility [38]. For instance, the meta-analysis by Lakatos *et al.* [174] showed that the abundance of ground nesting and feeding birds was negatively impacted by urbanization owing to higher predation by domestic animals as well as a lack of suitable nesting sites [174]. This meta-analysis also showed that migratory birds were most likely to avoid cities [174]. Cavity nesters have been shown to have a better chance of survival in cities owing to their ability to use artificial nesting sites such as nest boxes [175]. The relationship between health and species traits might simply be absent in the context of urbanization. Alternatively, this relationship may be dependent on the health biomarker measured. For example, insectivorous birds may be negatively impacted by urbanization owing to limited food sources and this may have consequences for specific aspects of health such as body condition or carotenoid levels, but we may not see an impact on overall health. Future studies could look at this specific question.

It would also be expected that impacts of urbanization on health may depend on the latitude of the study site, as there is latitudinal variation in rainfall, temperature and other climatic variables, and certain vector-borne diseases have been shown to be dependent on the climate [176]. For instance, in the bat *E. fuscus*, the negative impact of fungal infections became more prevalent at higher latitudes, possibly because warming winter conditions in northern latitudes may lead to insect declines reducing food availability for the bats [177]. However, we did not find any relationship between latitude, urbanization and health in our study. The majority of studies included in our meta-analysis were conducted at similar mid-to-high latitudes, and thus we had little power to detect a latitudinal relationship between urbanization and health, if this was present. There is a strong need for studies conducted at lower latitudes, particularly in tropical mega-cities where urbanization rate has been high in recent decades [39]. The lack of studies looking at the impacts of urbanization in the southern hemisphere is a massive gap in knowledge and is of particular importance as urbanization in the global south is rapidly increasing. The differences in environment and the fact that many developing countries are located in the south may mean we would see a different relationship between urbanization and health than what we are currently seeing in this meta-analysis [53].

We performed follow-up analyses on nestlings and adult birds separately because it is likely adult birds may be affected differently when living in urban environments than nestlings, as the developmental period is an extremely sensitive time, particularly when responding to environmental stressors [31–33]. Our results show a strong tendency for urban adults to have higher levels of blood glucose than non-urban adults, although this difference was not significant. This could indicate fluctuations in the availability and quality of food found in urban areas as anthropogenic food that is widely available in urban environments is usually found to be higher in sugars than natural food sources and lead to an energetic imbalance when consumed [178,179]. Higher glucose levels may also indicate that urban birds have higher metabolic demands than non-urban birds. Urban birds have been shown in a variety of studies to have increased levels of activity at the night, which has been attributed to increased exposure to artificial light [180–182]. This increase in activity can lead to an increased demand for energy, in birds, this energy supply will mainly come from glucose [183]. The relationship we found in our meta-analysis was only based on a small sample size, therefore there is an increased need for more studies focusing on the metabolic demands that urban birds face, as this could constrain them when it comes to other energetically demanding activities including reproduction.

We also show that urban nestlings are in poorer body condition when compared to non-urban nestlings, but adult body condition did not differ between habitat types. One thing to note is that we did find evidence of time lag bias in the nestling data subset, this may indicate that studies within this subset with significant results may have been published earlier than those without significant results. The most likely cause of nestlings being in poorer body condition in urban environments is reduced food availability or quality. Nestlings that are raised in urban environments are more likely to be fed with poor-quality food that may lack the nutrients required for development, as shown in a variety of bird species [36,89,184]. The availability of good-quality food is important for both chick growth and condition which is shown in the meta-analysis [185]. The fact that we see a negative relationship between urbanization and health in nestlings but not adults could be seen as surprising as conditions during the development period have been found to have long-lasting effects into adulthood [186,187]. However, this result may be a consequence of the selective disappearance of birds that are in poorer condition before they reach adulthood in urban areas. This has been shown in great tits, where juvenile individuals with shorter telomeres disappeared from the urban population [51]. Alternatively, the favourable winter conditions in urban areas, with availability of anthropogenic food and higher temperature compared with rural areas, may allow birds to recover from the stressors encountered during their developmental period, and thus increase winter survival and health [162].

We also found that the negative effects of urbanization on body condition of nestlings was dependent on their primary behaviour, bird species with specialist behaviours including those classed as terrestrial were shown to be in poor body condition, whereas generalist species were not impacted by urbanization. Much of the literature indicates that an organism’s ability to succeed in an urban environment will depend on them having specific traits that would allow them to take advantage of their surroundings. Species that are classified as ‘generalists’ can be more flexible to changing conditions including land modification, as they are able to adapt to a wide range of both habitat and feeding conditions [188]. Species that specialize in certain behaviours do not have this luxury and are more likely to be negatively impacted by modifications to their habitat [189]. For example, ‘terrestrial’ bird species spend most of their time on the ground where they will feed and nest, however, this behaviour makes them more vulnerable to urban stressors including predation by domestic cats and dogs as well as habitat modification or degradation caused by urban activities as shown in Lakatos *et al.* [174] meta-analysis [174]. Nestlings that are in worse body condition and weigh less are less likely to successfully fledge and have a lower survival rate [190]. Thus, our results point to the early life exposure to urban stressors being a considerable challenge for urban bird populations. However, the demographic consequences of poor body condition and lower survival of urban nestlings are vastly overlooked, but it is essential that future studies quantify such effects to improve our understanding of whether or not urban bird populations are self-sustained [191,192].

## 5. Conclusions

Our meta-analysis contradicts the popular belief that urbanization is generally bad for the health of wild birds. As health is a complex and multi-variate trait, different components of health will be impacted in different ways by urbanization. To reinforce this idea, we showed that using a continuous rather than a dichotomous descriptor of urbanization can improve the inference and does suggest an overall negative relationship between urbanization and health. However, the effect size was rather small and only visible when the degree of urbanization was very high. Moreover, heterogeneity was very high, highlighting that any effect may depend on the species studied, the life stage, the location and the measure of health used. We highlight the importance for future studies to measure the level of urbanization as this could impact the strength and direction of the relationship with health.

We also highlight that the body condition of nestlings seemed to be negatively affected by urbanization, which possibly points to lack of nutrients during growth as a key challenge in urban areas which may have negative fitness consequences. It is therefore clear that consideration must be taken to improve the body condition of birds during development. Planners could implement habitat improvement strategies aimed at increasing food resources for example insect abundance. The outcome in terms of insect availability and avian fitness could then be easily monitored. It is crucial that future studies will investigate what specific environmental factors may be more important for avian health, for example sensory pollutants including noise and light pollution, access to anthropogenic food or lack of suitable habitat. This information could be pivotal for the management of urban environments to ensure the future conservation of the species that inhabit them and allow for a more targeted conservation management approach.

## Data Availability

The authors of this work are committed to ensuring that data used in our analysis is made publicly available. The data and code used in this analysis are available on Zenodo [193]. Supplementary material is available online [194].
